# Study on dynamic expansion and β-carotene accumulation in storage root of orange-fleshed sweetpotato, and screening of germplasm resources with high rate of medium-sized storage roots

**DOI:** 10.3389/fpls.2026.1744016

**Published:** 2026-02-05

**Authors:** Guang-Yuan Ma, Cai-Yue Yan, Huilan Liu, Yong-Xian Chen, Zi-Han Zhao, Dao-Bin Tang, Kai Zhang, Ji-Chun Wang

**Affiliations:** 1 College of Agronomy and Biotechnology, Southwest University, Chongqing, China; 2 Chongqing Key Laboratory of Biology and Genetic Breeding for Storage Root (SR) and Root Crops, Chongqing, China; 3 Engineering Research Center of South Upland Agriculture, Ministry of Education, Chongqing, China; 4 Chongqing Beibei germplasm bank of Storage Root (SR) and root crops, Chongqing, China

**Keywords:** medium-sized storage root rate, OFSP, storage root expansion, sweetpotato, β-carotene

## Abstract

As an important food and economic crop, sweetpotato (*Ipomoea batatas* L.) has garnered widespread attention globally. Storage roots (SRs) of orange-fleshed sweetpotato (OFSP) varieties are rich in β-carotene, which holds significant importance for human health. The medium-sized SRs are popular in the market. However, germplasm resources with both high β-carotene content and medium-sized storage root rate (MSR) are severely lacking. This study analyzed SR expansion and β-carotene accumulation in six sweetpotato germplasm resources with varying β-carotene levels, and conducted a two-year dense planting field trial to boost MSR and screen elite OFSP germplasms with high β-carotene content, MSR and yield. Results revealed germplasm-specific differences in SR expansion and β-carotene accumulation. Among the tested germplasms, Qingyushu No.6, Yushu615 and Yuhongxinshu No.3 maintained stable β-carotene levels at about 9.09 mg/100g, 4.56 mg/100g and 11.54 mg/100g during growth, while Yuhongxinshu No.2 showed a zigzag rising trend from 4.99 mg/100g to 7.25 mg/100g. Over the two years of dense planting, Qingyushu No.6, Yushu615 and Yuhongxinshu 26 all achieved the MSR of over 70%, with the highest of 87.13%, indicating high MSR could be successfully obtained through dense planting strategy. Notably, superior germplasm, Yuhongxinshu 26, which simultaneously showed high β- carotene, MSR and yield, were successfully screened. These findings provide new insights into the physiological mechanisms and genetic potential of important traits in sweetpotato, offer theoretical support for optimizing cultivation practices, and lay theoretical and material foundations for breeding superior fresh-eating OFSP varieties with both high nutritional and commercial values.

## 1 Introduction

Sweetpotato is a crucial global food and cash crop, holding a significant position in agricultural production worldwide. It has strong stress resistance and wide applications and can serve as both food raw material and feed, as well as industrial raw material (Kong and Li, 2011), thus possessing important economic value. Sweetpotato is nutritionally rich, containing a large amount of starch, soluble sugars, and essential amino acids for the human (He, 2008). Besides being consumed fresh, sweetpotato can also be processed into high-value-added products such as French fries, dried sweetpotato, vermicelli, and sweetpotato powder. Notably, OFSP has attracted much attention due to their high content of β-carotene.

Previous studies have found that biosynthesis of carotenoids in sweetpotato storage roots is an isoprenoid metabolism driven by the methylerythritol phosphate (MEP) pathway in plastids. The direct precursor of carotenoid synthesis is Geranylgeranyl pyrophosphate (GGPP), which is catalyzed by enzymes to produce lycopene and further β-carotene catalyzed by lycopene β-cyclase (LCYB) (Noh et al., 2020). β-carotene is a plant pigment and a member of the carotenoid family. The long-chain conjugated double bond structure in the β-carotene molecule can absorb the blue wavelength in visible light, giving it an orange appearance. In OFSP, β-carotene accounts for 90% of the total carotenoids. It not only has important health functions but also significantly improves the nutritional quality of sweetpotato (Zhang et al., 2003). Van Jaarsveld et al. (2005a, b) found that consuming OFSP can significantly improve vitamin A deficiency in primary school students in South Africa. Based on this, the United Nations has included the consumption of OFSP in important programs to address vitamin A deficiency in African children and women.

OFSP is widely popular in the market due to their soft, waxy, sweet taste, bright color and rich nutritional value. Currently, sweetpotato breeding is shifting from solely pursuing high yields to focusing on high-quality and specialized traits. OFSP varieties with moderately sized storage roots, attractive shapes, and excellent taste are more favored in the market, commanding higher sales values and greater market potential. MSR, indicating the ratio of medium-sized SRs (the weight of each SR is between 50 to 250 g) in all storage roots (Stathers et al., 2019), thus become a new indicator for commercial value of OFSP.

Previous studies have found that the synthesis and accumulation of β-carotene are not only related to the gene expression regulation, but also closely associated with its sink structure and capacity (Chen et al., 2005). The color of sweetpotato flesh gradually deepens from white, milky white to yellow, and orange as the carotenoid content increases (Lin et al., 1989). Different soil types have a significant impact on the β-carotene content in sweetpotato SRs, and calcareous purple skeletal soil is more conducive to the accumulation of β-carotene in OFSP (Ding, 2018). Studies on OFSP has primarily focused on the effects of different environmental conditions and growth stages on carotene content (Caliskan et al., 2007), methods for carotene determination and extraction (Kimura et al., 2007), as well as the genotype-environment interaction effects of carotene (Manrique and Hermann, 2002). Li et al. (2010) selected five sweetpotato varieties with different flesh colors for reciprocal grafting and found that after grafting, the changes in β-carotene contents in SRs are jointly affected by rootstock, scion, growth period, and their interactions. Li et al. (2010) showed that the changes of β-carotene content in storage roots during the growth period can be divided into two types: significant change (Type i) and non-significant change (Type II). The study by Zhang et al. (2012) indicated that during the storage root diameter expansion period, the variation trend of β-carotene distribution in different parts of the storage root is basically consistent, and the β-carotene content in different growth stages mostly shows a positive correlation.

The development of sweetpotato fibrous roots into SRs can be roughly divided into the primary cambium active stage and the secondary cambium active stage. In the early stage, the primary cambium differentiates between the phloem and xylem and inhibits the lignification of the stele, determining whether the fibrous root can develop into a SR. In the late stage, the secondary cambium produces secondary xylem and parenchyma cells inward through cell division, and forms secondary phloem outward, promoting the rapid expansion of SRs (Firon et al., 2013; Miao et al., 2019; Qiu, 1960; Song et al., 2023). Previous study showed that, in OFSP, the carotenoid content in SRs shows a significant positive correlation with their fresh weight, dry weight, and the distribution ratio of photosynthates in SRs, while it exhibits a negative correlation with the distribution ratio of photosynthates in leaves and petioles (Li et al., 2010). For OFSP, based on variation trend, the variation patterns of carotenoids during the growth period can be divided into three types: overall stable type, continuous increase type, and tortuous rise type (Hou et al., 2013). In addition, the daily accumulation of carotenoids is negatively correlated with the accumulation of fresh weight, dry weight, starch, and other substances in SRs.

Previous study had found that there is a significant negative correlation between β-carotene and quality traits such as starch content of storage root (Zhang et al., 2016). This indicates that it is difficult to breed sweetpotato varieties with both high carotenoids and starch yield. The SR weight of Yanshu 25 and Pushu 32 showed an upward trend with the increase of growing days, reaching a relatively high level at 160 and 180 days after planting. This may be due to the fact that photosynthates are preferentially supplied to the growth of stems and leaves in the early growth stage, and mainly transported to SRs in the later stage (Zhao, 2022). A study by Li et al. (2017) also showed that within 0–70 days after transplanting, photosynthates are mainly supplied to the growth of aboveground stems and leaves, and after 70 days, they are mainly transported to the underground part to promote starch formation. The development of sweetpotato SR is characterized by first elongating and then thickening; in the late growth stage, the proportion of large and medium-sized SRs increases while that of small SRs decreases. The number of SRs per plant first increases and then decreases with the extension of the growth period (Yan et al., 2019). As an essential condition for photosynthesis, light directly affects the yield of aboveground assimilates and their distribution ratio to SRs, thereby determining SR yield (Na-Ra Lee and Lee, 2015). The leaf area, photosynthetic efficiency, and light conditions of sweetpotato directly affect their normal growth and development as well as the formation and accumulation of dry matter. Under low-light conditions, the photosynthetic rate of sweetpotato leaves decreased significantly, resulting in a reduction in the total amount of carbohydrates (e.g., glucose, starch) produced. The allocation priority of photosynthetic products was altered: the plant prioritized supplying limited nutrients to the aboveground vines for growth, in an effort to “pursue” more light, while the nutrients delivered to the underground tubers were substantially reduced. This led to the phenomenon of “uncontrolled vine growth” and stagnation in tuber enlargement (Li et al., 2023), sweetpotato vines will grow excessively, which inhibits the differentiation and expansion of SRs (Wang et al., 1985).

The growth cycle of sweetpotato includes physiological processes such as stem and vine elongation, increased branching, photosynthate translocation, SR enlargement, and dry matter accumulation. Studies have shown that there is a negative correlation between yield and starch content among sweetpotato varieties: varieties with rapid enlargement and high yield usually have lower starch content, while those with slow enlargement and low yield have higher starch content (Tang et al., 2014). Differences may exist in the patterns and magnitudes of nutrient accumulation among different varieties, which in turn affect yield traits (Chen et al., 2004). The formation and expansion of sweetpotato SRs are regulated by the aboveground and underground resource sinks, and are also susceptible to environmental conditions (Yan et al., 2019). Therefore, appropriate cultivation measures can be adopted for sweetpotato to achieve the desired SR size.

There are differences in SR characteristics, such as size, enlargement patterns, β-carotene accumulation and content among different sweetpotato varieties, which may be closely related to factors such as the genetic background of the variety, growth environment, and cultivation management. At present, there is a lack of in-depth understanding of the laws of SR expansion and β-carotene accumulation during the SR development process of OFSP. Moreover, there is a scarcity of germplasm resources with high carotene content and a high MSR (above 55%), which seriously hinders the breeding process of high-quality OFSP varieties. As a result, there is a severe shortage of OFSP varieties with high carotene content, high MSR, and high yield that can meet market demand.

In this study, sweetpotato germplasm resources with different carotene contents were selected to investigate the laws of OFSP expansion and carotene accumulation during their SR development. Additionally, a two-year field dense planting experiment was conducted to improve the MSR, and orange-fleshed germplasm resources with a high MSR were screened. This research lays a theoretical and material foundation for breeding OFSP varieties with high carotene content and further enhancing the commercial value of fresh-eating sweetpotato.

## 2 Materials and methods

### 2.1 Plant material and overview of the test location

A total of six range-fleshed germplasm resources, Qingyushu No.6 (Z1), Yuhongxinshu 34 (Z2), XS160615 (Z3), Yuhongxinshu 26 (Z4), Yuhongxinshu No.3 (Z5) and Yuhongxinshu No.2 (Z6), were used as test materials. Regularity of SR expansion and β-carotene accumulation were detected in Z1, Z3, Z5, Z6; Dense planting experiments were conducted using Z1 to Z6 in 2022 and 2023. Over the two years, Ningzishu No. 1 was used as the control (CK). Potassium sulfate compound fertilizer (N:P_2_O_5_:K_2_O=15:15:15) was applied 30kg·667m^-2^ during the experiment. The experiment was conducted at the sweetpotato Research Base in Xiema Town, Beibei District, Chongqing (longitude 106.37°E, latitude 29.76°N, altitude 600 m), belonging to the subtropical monsoon humid climate. The annual average temperature was 20.5°C in 2022 versus 19.8°C in 2023, with high average temperature during June to August as 35°C in 2022 and 30°C in 2023, respectively. Annual precipitation was 950 mm in 2022 and 1200 mm in 2023. The test soil was a weakly alkaline soil with a pH of 8.1, containing 1.31 g·kg^-1^ of nitrogen (N), 0.96 g·kg^-1^ of phosphorus (P), and 25.10 g·kg^-1^ of potassium (K).

### 2.2 Detection of the regularity of SR expansion and β-carotene accumulation the in OPSP

A randomized block design was adopted with three replications. Using the commonly used density in production, each plot had an area of 5 m × 0.9 m = 4.5 m², with 24 plants planted per plot, plant spacing 17.5 cm, line spacing 75.6 cm, resulting in a planting density of 53,300 plants per hectare. SR sampling and investigation began at 50 days after planting (DAP). For each germplasm resource, three plants were sampled every 15 days, i.e., a total of 7 samplings were conducted at 50, 65, 80, 95, 110, 125, and 140 DAP. The SR weight per plant was weighed, β-carotene was measured according to the method described previously (Zhang et al., 2016). Briefly, the samples were washed and peeled, and approximately 1.0 g was chopped into small pieces, milled and extracted with 5 mL of acetone repeatedly until no color remained in the extract. The extracts were transferred to a 50 mL capacity bottle and centrifuged at 2500 r/min for 5 min. The absorbance of the supernatant at 454 nm was measured and the β-carotene content was calculated by comparison with an external standard curve.

The time when SR started to expand was observed, and the SR weight per plant were measured to calculate the root SR enlargement rate (RTR) as:


$$\text{RTR}=\frac{\text{W}(\text{n}+1)-\text{Wn}}{15}$$

That is, the difference in dry weight of root SRs per plant between two samplings divided by the number of interval days (where W represents the SR weight per plant and n represents the number of samplings) (Hou et al., 2015).

### 2.3 Screening of high MSR germplasm resources through dense planting strategy

Aiming to obtain high MSR and screening of high MSR germplasm resources through dense planting strategy, the dense planting cultivation measure was adopted using the double-row zigzag planting method on a single ridge. In 2022 and 2023, with Ningzishu No. 1 as the control (CK), a randomized block experiment was conducted with three replications. Each plot had an area of 5 m × 0.9 m = 4.5 m², and 34 seedlings were planted per plot, plant spacing 17.5 cm, line spacing 75.6 cm, resulting in a planting density of approximate 75,600 plants per hectare. Routine field management was performed, and the SRs were harvested on November 2, 2022 and October 23, 2023, respectively. Ten plants were randomly harvested from each plot, and according to the description by market trends and reference (Stathers et al., 2019), the SRs were classified into large-sized SRs (250 g and above), medium-sized SRs (50–250 g), and small-sized SRs (below 50 g). The number and fresh weight of each category were counted, and the medium SR rate and converted yield (kg·hm^-^²) were calculated. Three medium-sized SRs were taken from each plot, and the β-carotene content in the SRs was determined according to the method described in 1.2. The large-sized SR rate, MAR, and small-sized SR rate was determined as the proportion of large-sized SR, medium-sized SR and small-sized SR weight to the weight of all SRs (%), respectively.

### 2.4 Statistical analysis

All data were presented as means ± standard deviations of at least three independent determinations on one sample for each period. One-way ANOVA and multiple comparison tests were conducted using SPSS version 18.0 (IBM, Armonk, New York, USA). Statistical significance was established at *P* < 0.05.

## 3 Results and analysis

### 3.1 Accumulation pattern of β-carotene in SRs of OFSP germplasms

**Figure 1:**
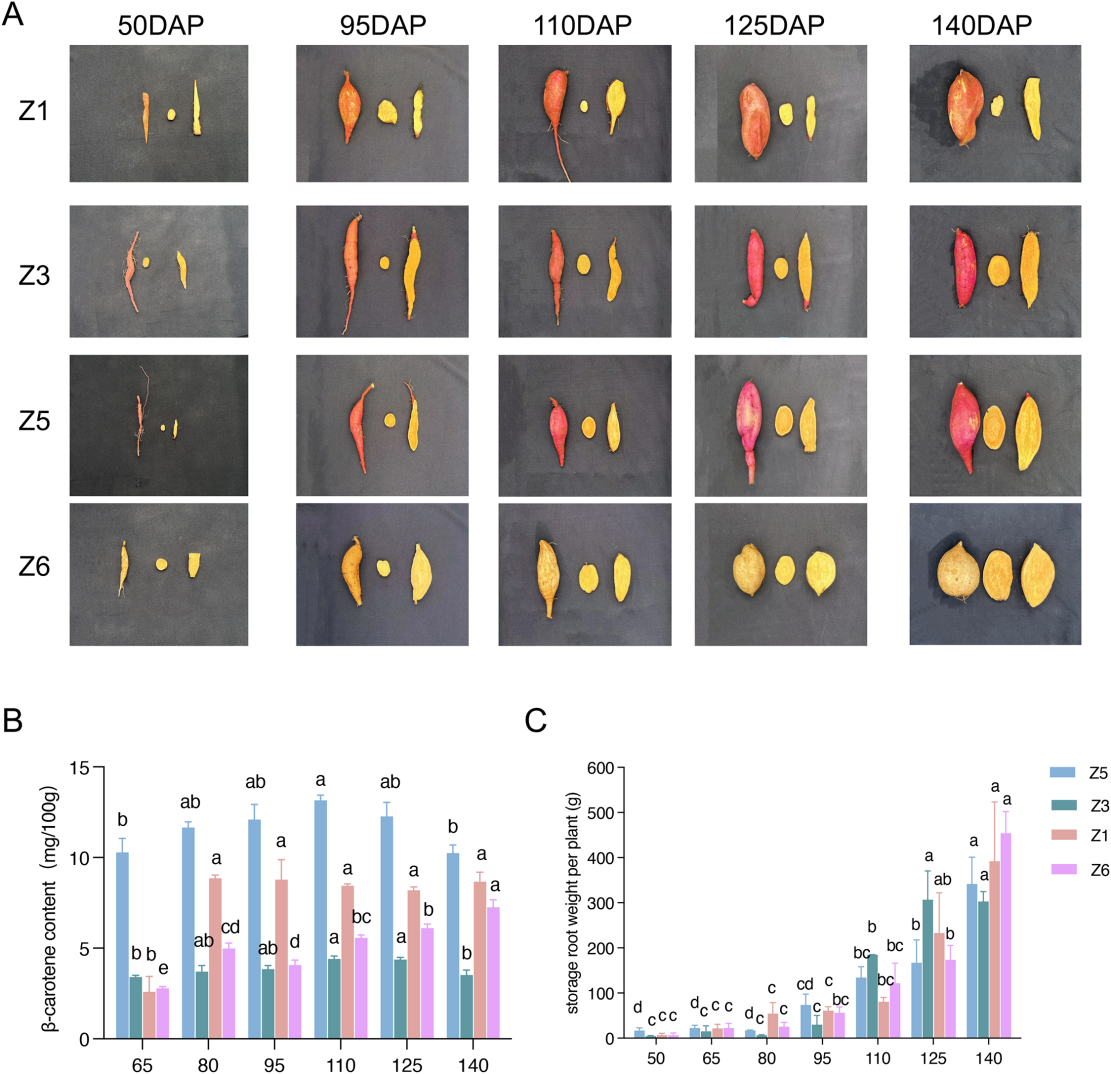
SR expansion and β-carotene accumulation during SR development in orange-fleshed sweetpotato germplasms. **(A)** Changes of sizes and flesh colors during the developments of SRs in different germplasm resources. **(B)** Changing patterns of β-carotene content accumulation in storage roots of tested germplasm resources. **(C)** Dynamic changes of SR weight at different developmental periods in the tested germplasm resources. Different lowercase letters indicate significant differences at the P< 0.05 level.

The tested germplasm resources showed different SR-setting characteristics and β-carotene contents in SRs. To clarify the accumulation pattern of β-carotene in germplasm resources of OFSP, sampling investigations were conducted starting from 50 DAP, with samples taken every 15 days until 140 DAP, and the degree of SR expansion and β-carotene content were measured. As shown in **Figures 1A, B**, the SR β-carotene content of Z1 increased significantly to about 9.09 mg/100g at 80 DAP and then stabilized, the β-carotene content of Z3 remained stable in the early stage of SR expansion at about 4.56 mg/100g but decreased at 140 DAP. For the tested sweetpotato germplasm Z5, there was no significant difference in the β-carotene content of SRs at 11.540 mg/100g between each sampling time and the previous one. The β-carotene content of Z6 showed significant increases at 80, 110, and 140 DAP, from 4.99 to 5.57 mg/100g and finally up to 7.25 mg/100g, respectively. According to the classification method proposed by Hou et al. (2013), the β-carotene variation types of in the tested OFSP materials Z1, Z3, and Z5 during the growth period were classified as the overall stable type, while Z6 belonged to the zigzag rising type. Analysis of variance showed that there were significant differences in the β-carotene content of SRs among different developmental stages (*P* < 0.05).

### 3.2 Study on the expansion pattern of SR and β-carotene accumulation in OFSP

Significant differences in SR weight could be detected among developmental stages (**Figure 1C**, **Table 1**). The RTR of Z1 showed significant increases at 10.15g·d^-1^ and 10.62g·d^-1^ during 110–125 and 125–140 DAP, respectively, while the peak period of SR expansion for Z3 was from 95 to 110 DAP, with RTR of 10.34g·d^-1^, after which the RTR gradually decreased to 0.07g·d^-1^. Detection on RTR showed that Z5 and Z6 had two peak periods of SR enlargement. The first peak occurred from 95 to 110 DAP, with the RTR of 4.06g·d^-1^ and 4.38g·d^-1^, respectively, then followed by a trough period of expansion from 110 to 125 DAP and then the second peak period of 125–140 DAP, shown as the RTR of 11.65g·d^-1^ and 18.69g·d^-1^, which higher than that in the first peak period (**Table 1**). According to the results of variance analysis, there was significant difference (*P* = 0.016) among different developmental stages but no significant difference (*P* = 0.845) among germplasms, indicating the OFSP germplasms showed similar SR expansion patterns.

**Table 1:** RTR of storage roots(g.d^-1^).

Germplasm	50–65 DAP	65–80 DAP	80–95 DAP	95–110 DAP	110–125 DAP	125–140 DAP
Z1	0.97 ± 0.85cd	2.20 ± 1.92bcd	0.43 ± 2.25d	1.32 ± 0.56cd	10.15 ± 9.17a	10.62 ± 6.82ab
Z3	0.71 ± 0.67cd	0.17 ± 0.14d	0.80 ± 1.79d	10.34 ± 2.36a	7.79 ± 3.88abc	0.07 ± 2.45d
Z5	0.38 ± 0.12d	0.09 ± 0.18d	3.32 ± 2.11cd	4.06 ± 1.21bc	2.19 ± 4.66cd	11.65 ± 4.26ab
Z6	0.86 ± 0.72cd	0.21 ± 0.14d	2.03 ± 0.76bcd	4.38 ± 3.73bc	3.44 ± 2.79bcd	18.69 ± 5.64a

Different lowercase letters indicate significant differences at the *P* < 0.05 level.

### 3.3 Screening and identification of sweetpotato germplasm resources with high MSR

**Figure 2:**
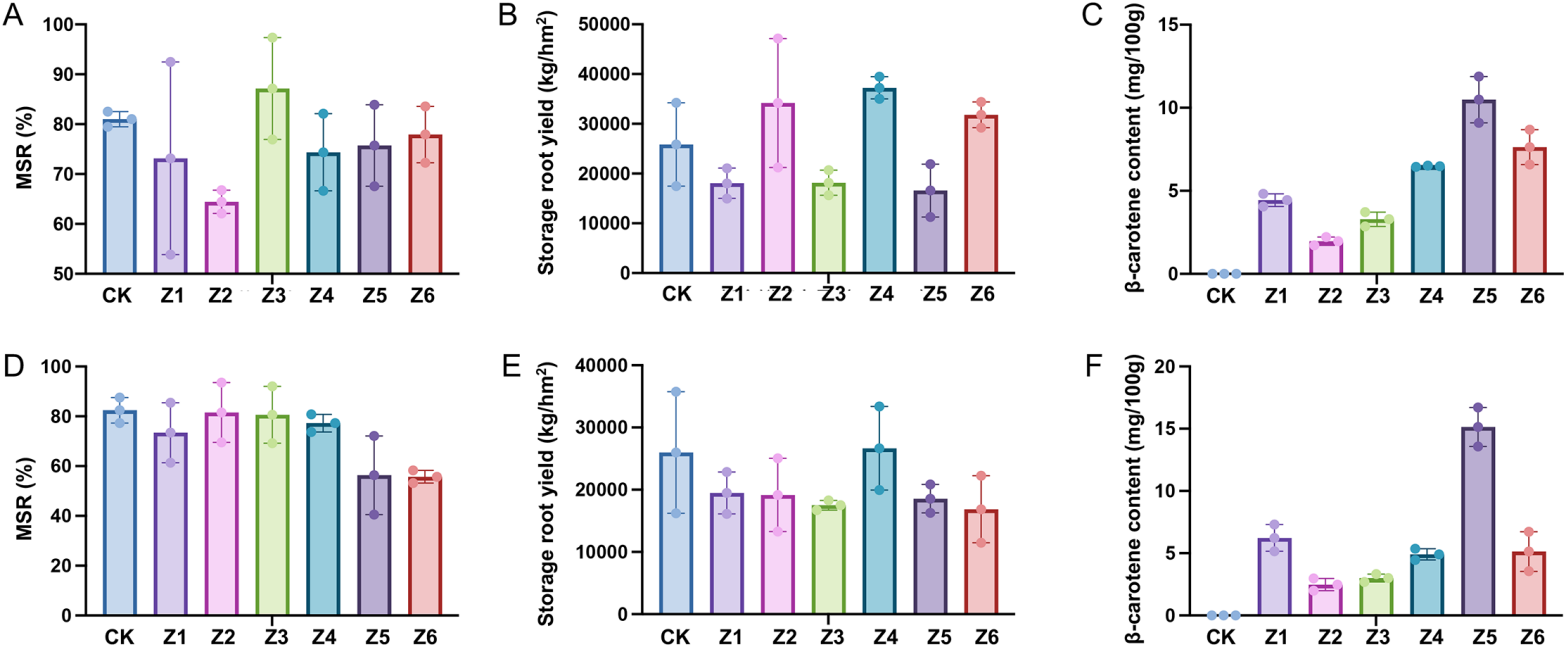
Determination of the MSR, yield and β-carotene content in the dense planting trail in 2022 and 2023. **(A–C)**, determination of the MSR, yield and β-carotene content in 2022, respectively. **(D–F)**, determination of the MSR, yield and β-carotene content in 2023, respectively.

Field trails of the MAR of the tested OFSP materials were conducted through dense planting strategy. In 2022, during the harvest stage, the number and weight of large, medium, and small-sized SRs as well as the β-carotene content were measured, respectively, and the equivalent yield (kg·hm^-^²) was calculated based on the total SR weight and transplanting density, the results were shown in **Figure 2** and **Table 2**.

**Table 2:** Results of two-years dense planting trail.

Year	Germplasm	Large-size storage root rate (%)	Medium-size storage root rate (%)	Small-size storage root rate (%)	Fresh SR yield (kg hm^-2^)	β-carotene content (mg 100g^-1^)
2022	CK	5.27 ± 9.13b	81.00 ± 1.52ab	13.73 ± 7.65ab	25849.85 ± 8358.57ab	–
	Z1	10.50 ± 18.19ab	73.16 ± 19.3ab	16.34 ± 5.17a	18015.98 ± 3029.3b	4.44 ± 0.38
	Z2	30.94 ± 3.73a	64.43 ± 2.33b	4.63 ± 1.78cd	34178.82 ± 12946.77a	1.97 ± 0.25
	Z3	10.72 ± 9.73ab	87.13 ± 10.21a	3.22 ± 1.02d	18159.98 ± 2534.14b	3.29 ± 0.43
	Z4	12.93 ± 8.34ab	74.37 ± 7.74ab	12.70 ± 2.31abc	37244.30 ± 2242.6a	6.48 ± 0.04
	Z5	12.76 ± 12.1ab	75.74 ± 8.17ab	11.50 ± 4.37abcd	16586.96 ± 5309.28b	10.49 ± 1.39
	Z6	16.13 ± 5.35ab	77.92 ± 5.66ab	5.96 ± 1.32bcd	31805.35 ± 2590.28a	7.63 ± 1.05
2023	CK	6.36 ± 11.02b	82.44 ± 5.12a	11.20 ± 7.61b	25977.32 ± 9768.86a	–
	Z1	6.09 ± 10.55b	73.43 ± 12.05ab	20.48 ± 9.31b	19492.20 ± 3361.08a	6.23 ± 1.08
	Z2	5.83 ± 10.10b	81.57 ± 12.00a	12.60 ± 6.82b	19152.00 ± 5885.20a	2.48 ± 0.49
	Z3	3.40 ± 5.90b	80.66 ± 11.42a	15.94 ± 5.95b	17501.40 ± 781.10a	3.01 ± 0.32
	Z4	7.82 ± 6.84b	77.25 ± 3.52a	14.93 ± 3.57b	26661.60 ± 6716.21a	4.91 ± 0.45
	Z5	0.00 ± 0.00b	56.35 ± 15.77b	43.65 ± 15.77a	18559.80 ± 2283.07a	15.14 ± 1.57
	Z6	32.58 ± 10.26a	55.74 ± 2.54b	11.69 ± 8.39b	16858.80 ± 5378.24a	5.14 ± 1.59

Different lowercase letters indicate significant differences at the *P* < 0.05 level.

#### 3.3.1 Identification of MSR in tested OFSP germplasms in 2022

The results showed that through dense planting, after calculating the MSR following the 2022 harvest, it was found that except for Z2, whose medium-SR rate was lower than 70% at 64.63% due to an excessive number of small SRs, the MSR of the other five tested germplasm resources were all higher than 70% at Z1 (73.16%), Z3 (87.13%), Z4 (74.37%), Z5 (75.74%), Z6 (77.92%).

#### 3.3.2 Identification of MSR in tested OFSP germplasms in 2023

In 2023, the MSRs of Z5, Z6 did not reach 70%, while the MSRs of the remaining 4 germplasm resources were all higher than 70%. Among them, Z5 had no large SRs in all three replicates, its low MSR at 56.35% was likely due to a relatively large number of small SRs at 43.65%; in contrast, Z6 had lower MSR at 55.74% because of a relatively large number of large SRs at 32.58%. This indicates that different OFSP germplasm resources have different SR-setting characteristics and expansion patterns. Based on the combined results of the two-year experiments, the germplasm resources with MSR reaching over 70% in both years were Z1, Z3, and Z4.

#### 3.2.3 Analysis of yield of test OFSP germplasm resources

In 2023, there was no significant difference among the tested OFSP germplasm resources. In 2022, the fresh yield of Z1 (18015.980kg·hm^-2^), Z3 (18159.980kg·hm^-2^) and Z5 (16586.960kg·hm^-2^) were significantly lower than that of other germplasm resources, indicating that the production potential of these germplasms might be influenced by environment. The fresh yields of Z4 (37244.300kg·hm^-2^) were higher than CK (25849.850kg·hm^-2^) and most of other germplasms in both of two years, indicating its relatively excellent production performance and strong environmental adaptability, as well as its high yield potential under dense planting condition.

Based on the comprehensive analysis of the two-years experiment, Z4 not only achieved a high MSR, but also showed a high yield under dense planting conditions. This indicates that it may have a good application prospect in the future high-yield dense planting sweetpotato cultivation.

## 4 Discussion

### 4.1 Variations in β-carotene content and expansion patterns of different sweetpotato germplasms

Our results revealed that there were differences in β-carotene accumulation and SR enlargement patterns during SR development among different OFSP germplasm resources. Hou et al. (2013) found the β-carotene content of sweetpotato variety Xuyushu 34 increased rapidly from 40 to 70 DAP, reaching a maximum value of 29.610 mg.kg^−1^ at 70 DAP. Subsequently, from 70 DAP until harvest, the β-carotene content decreased slowly, showing a unimodal fluctuation curve of “increase ~ decrease” throughout the growth period. Zhong et al. (2023) also found a similar situation on the sweetpotato variety Pushu 17, where the β-carotene content showed a fluctuating curve of “increase ~ decrease” during growth period. The difference is that the β-carotene content of this variety reached the maximum at 90 DAP. Peiyong et al. (2014) evaluated three sweetpotato varieties, Ning 4-6, Sushu 20 and Sushu 8, and found that the dynamic changes of β-carotene content in the SRs varied with varieties, but all showed a significant decrease after 120 DAP. The above studies indicate that there were certain differences in carotene content among different sweetpotato germplasm resources with the change of growth period.

In this study, during the investigation from 50 to 140 DAP, it was found that different sweetpotato germplasms showed different trends in terms of β-carotene content changes. Specifically, the β-carotene content of Z3 decreased significantly at 140 DAP, while that of Z1 increased significantly at 80 DAP and then tended to be stable. The β-carotene content of Z6 increased significantly at 80, 110, and 140 DAP, the β-carotene change types of Z1, Z3, and Z5 were of the overall stable type, while Z6 shows a tortuous upward type. It was found that the β-carotene content in the storage roots of the tested germplasm resources Z3 and Z5 also showed a similar “increase ~ decrease” trend, but the β-carotene content of Z1 and Z6 showed an overall continuous upward trend. These results indicated that the accumulation regularity of β-carotene varied among germplasm resources.

Differences are also observed in the RTR among different sweetpotato germplasm resources. A study on Qinshu 4 revealed that the SR expansion process exhibits two distinct peak periods. The first peak period of enlargement occurs 80 to 100 DAP, followed by a slowdown in RTR during 100 to 120 DAP, entering a trough period. The second peak period of enlargement appears 120 to140 DAP, with an RTR significantly higher than that of the first peak period. At this stage, the growth of stems and vines slows down significantly, and a large amount of photosynthate is transported to the storage roots, making it a critical period for the accumulation of storage root dry matter (Hou et al., 2015). In this study, it was found that the second peak period of enlargement for Z5 and Z6 occurs after 125 DAP, while the enlargement peak period of Z3 is between 95 to 110 DAP, after which the RTR gradually decreases; the enlargement peak period of Z1 was after 110 DAP, with the RTR showing an increasing trend. This is generally consistent with the previous studies indicating that sweetpotato start to enlarge rapidly after 90 DAP (Zhang et al., 2012; Shen et al., 2020). These results suggested that in actual production, targeted cultivation measures should be adopted according to the SR development and β-carotene accumulation patterns of different varieties, and also should be carried out during the critical periods of storage root enlargement and carotene accumulation to promote the improvement of yield and β-carotene content.

Our study revealed different characteristics of SR enlargement and β-carotene accumulation process in different OFSP germplasm resources, provide a new perspective for unraveling the physiological mechanisms and genetic potential of SR morphology and quality formation in sweetpotato, and offer theoretical support for optimizing cultivation and management measures.

### 4.2 Effects of dense planting on the MSR of OFSP germplasms

The results of field identification on the MSR showed that dense planting cultivation measures can achieve a very high MSR in OFSP. Among the tested germplasm resources in 2022, five germplasms reached a MSR of over 70%, and in the 2023 experiment, four germplasm resources achieved a MSR of more than 70%. Such a high MSR is very difficult to reach in actual production, indicating that dense planting can effectively improve the MSR of sweetpotato. In addition, three germplasm resources maintained a MSR of over 70% in both years, while Z5 failed to reach 70% in both years due to an excessive number of small SRs. It was suggested that the MSR was related to the SR-setting characteristics of the germplasm itself and was influenced by the genotype. These results indicated that the MSR of fresh-eating sweetpotato can be improved through cultivation measures, but different sweetpotato genotypes may show varying response to these cultivation measures. It is necessary to formulate corresponding cultivation measures based on the characteristics of sweetpotato varieties to effectively improve the traits such as MSR, thereby meeting market demand and increasing economic benefits.

Based on the results of a two-year experiment, the germplasm resource Z4 has shown excellent performance in terms of the MSR and fresh yield, indicating that this germplasm resource has strong adaptability and high production potential under different environmental conditions. Specifically, the MSR of Z4 remains at a high and stable level, and it has outstanding performance in yield, which further proves its important application value in the breeding of fresh-eating OFSP. Germplasm resources with high carotene content, high MSR and high yield have both high nutritional and market values, and have broad market prospects. They can be used as elite materials for the improvement and breeding of fresh-eating sweetpotato varieties.

In conclusion, through dense planting, the MSR of sweetpotato can be increased to over 70%, confirmed the application prospect of dense planting strategy in sweet potato production to achieve a high MSR. Furthermore, a series of germplasm resources with both high nutritional value and high commercial value were screened out, which can be used as recommend fresh-eating OFSP varieties in the market or parental materials for superior variety breeding. This study provides a scientific basis for variety improvement and cultivation technology development of sweetpotato, lays a solid theoretical and material foundation for “high-yield, high-quality, and high commercial value” sweetpotato production.

## Data Availability

The raw data supporting the conclusions of this article will be made available by the authors, without undue reservation.

## References

[B1] Caliskan M. E. Sogut T. Boydak E. Erturk E. Arioglu H. (2007). Growth, yield, and quality of sweetpotato (Ipomoea batatas (L.) Lam.) cultivars in the southeastern anatolian and east mediterranean regions of Turkey. Turkish J. Agric. Forestry 31, 213–228. doi: 10.3906/tar-0606-11

[B2] Chen Y. Yuan S. Q. Wang X. R. Tang A. J. Zhu W. B. (2004). The regularity of accumulation and distribution of dry matter in spring sweetpotato Qinshu No.4. Acta Agric. Boreali-Occident Sin. 13, 108–111.

[B3] Chen X. Y. Yuan Z. N. Zhang Z. J. Zheng Y. F. Zheng J. G. (2005). Isolation of total RNA from SRs of *Ipomoea batatas* and cloning of conserving domain sequence of *psy* gene. J.Fujian Agric. For Univ 34, 67–69.

[B4] Ding Y. (2018). Effects of soil species on growth, yield and quality of sweetpotato (Chongqing, China: MS Thesis of Southwest University), 43.

[B5] Firon N. Labonte D. Villordon A. Sela N. Dhekney A. Schaffer A. . (2013). Transcriptional profiling of sweetpotato (Ipomoea batatas) roots indicates down-regulation of lignin biosynthesis and up-regulation of starch biosynthesis at an early stage of storage root formation. BMC Genomics 14, 460. doi: 10.1186/1471-2164-14-460, PMID: 23834507 PMC3716973

[B6] He S. S. (2008). Processing utilization of sweetpotato. Farm Prod Process 5), 93–94.

[B7] Hou M. Zhang Y. G. Liu Y. J. Wang X. Tang W. Yan H. . (2015). Variation dynamics and mutual relationships of contents of main nutrients in expanding SRs of orange-flesh sweetpotato. Acta Agric. Jiangxi 27, 22–25.

[B8] Hou M. Zhang Y. G. Wang X. Tang W. Liu Y. J. Tang Z. H. . (2013). Variation laws of carotenoids content in storage root of orange-fleshed sweetpotato and its relationships with major economic traits. Sci. Agric. Sin. 46, 3988–3996.

[B9] Kimura M. Kobori C. N. Rodriguez-Amaya D. B. Nestel P. (2007). Screening and HPLC methods for carotenoids in sweetpotato, cassava and maize for plant breeding trials. Food Chem. 100, 1734–1746. doi: 10.1016/j.foodchem.2005.10.020

[B10] Kong Y. L. Li Q. C. (2011). Cultivation techniques of fresh edible sweetpotato variety. China Seed Industry 1), 64–66.

[B11] Li M. Fu Y. F. Wang D. Y. Tan W. F. Yang C. X. Zhang Q. T. (2010). Variations of β-carotene content in sweetpotato [*Ipomoea batatas* (L.) Lam] storage roots after reciprocal grafts among 5 varieties with different flesh color. Southwest China J. Agric. Sci. 23, 462–463.

[B12] Li C. Xue G. W. Huang J. Y. Wang N. D. Lu G. Q. (2017). Effects of different growth stages on nutritional components and processing characteristics of sweetpotato cultivar Xinxiang. Acta Agric. Zhejiangensis 29, 1957–1962.

[B13] Li X. Zhang Y. Wang L. Liu Y. Wang Y. Zhang Y. . (2023). Physiological and transcriptome responses of sweet potato [Ipomoea batatas (L.) Lam] to weak-light stress. Plant Physiol. Biochem. 191, 107345. doi: 10.1016/j.plaphy.2023.107345, PMID: 39204650 PMC11359650

[B14] Lin D. Y. Li W. Liu X. Y. (1989). Studies on carotenoid and flesh color of root SR in sweetpotatoes. Acta Agron. Sin. 15, 260–266.

[B15] Manrique K. Hermann M. (2002). Effect of G × E interaction on root yield and beta-carotene content of selected sweetpotato [Ipomoea batatas (L.) Lam.] varieties and breeding clones. ( International Potato Program Report) 281–287, CIP Program Report 1999-2000.

[B16] Miao X. R. Niu J. Q. Wang D. B. Liu Y. H. Li J. Y. Zhang J. . (2019). Research progress on regulation mechanism of formation and expansion of storage root and stem in root and SR crops. Mol. Plant Breed. 17, 2042–2047. doi: 10.7740/kcs.2015.60.6.491

[B17] Na-Ra Lee K.-H. C. Lee S.-Y. (2015). Effects of planting density and harvesting time on production of small-size tuberous roots in sweetpotato. Korean Crop Sci. 60, 491–497. doi: 10.7740/kcs.2015.60.6.491

[B18] Noh J. Kim J. Kim Y. Park S. C. Kim J. H. Kim Y. S. (2020). Transcriptome analysis reveals the MEP pathway and carotenoid biosynthesis genes involved in β-carotene accumulation in sweet potato storage roots. BMC Plant Biol. 20, 345. doi: 10.1186/s12870-020-02556-8 32698774

[B19] Peiyong M. A. Zhaodong J. I. A. Xiaofeng B. I. A. N. Xiaoding G. U. O. Yizhi X. I. E. (2014). Dynamic accumulation of β-carotene and dry matter in orange-fleshed sweetpotato [*Ipomoea batatas* (L.) Lam] and their correlation analysis. Agr Sci. Technol. 15, 1249–1252.

[B20] Qiu Z. G. (1960). The formation of sweetpotato storage roots. Bull. Biol. 07), 289–292.

[B21] Shen S. F. Xiang C. Wu L. H. Li B. (2020). A special case of pyramiding breeding of carotene content and dry matter content in sweetpotato. Mol. Plant Breed 18, 3032–3040.

[B22] Song W. H. Hou M. Zhang Y. G. Li J. Y. Liu Y. H. Wang Y. J. . (2023). Research progress on the mechanism of sweetpotato storage root expansion. Mol. Plant Breed. 4, 1–8.

[B23] Stathers T. Low J. Amagloh F. Carey T. Kyalo G. Agili S. . (2019). Handle with care: maintaining the quality and value of your sweetpotato roots during and after harvest through better practice. Int. Potato Center 10, 1–10.

[B24] Tang W. Li Q. Zhang Y. Wang X. Hou M. Ma D. (2014). Analysis on the essential characters and biological yield change of purple-fleshed sweetpotato Xuzishu 3. Agr Sci. Technol. 15, 1660–1666.

[B25] van Jaarsveld P. J. Faber M. Tanumihardjo S. A. Nestel P. Lombard C. J. Benadé A. J. (2005a). β-carotene–rich orange-fleshed sweetpotato improves the vitamin a status of primary school children assessed with the modified-relative-dose-response test. AJCN 81, 1080–1087. doi: 10.1093/ajcn/81.5.1080, PMID: 15883432

[B26] Van Jaarsveld P. Schippers J. H. M. Labuschagne M. T. Hugo A. de Kock H. M. (2005b). Carotenoid composition of South African sweetpotato cultivars. South Afr. J. Bot. 71, 471–478. doi: 10.1016/j.sajb.2005.03.003

[B27] Wang X. C. Liu B. X. Ji M. (1985). Anatomy of the root SR formation of sweetpotato varieties. Acta Agron. Sin. 11, 275–280.

[B28] Yan H. Zhang Y. G. Liu Y. J. Wang X. Hou M. Tang W. . (2019). Effects of growth stage on quality and SR traits of new sweetpotato cultivar Xuzishu8. Jiangsu J. Agric. Sci. 35, 9–14.

[B29] Zhang X. M. Mou D. H. Zhang S. S. Ma Z. M. (2012). Variations of β-carotene content in storage root expansion period of sweetpotato. Jiangsu Agric. Sci. 40, 303–305.

[B30] Zhang L. M. Wang Q. M. Wang Y. C. (2003). The main nutrient components and health care function of sweetpotato. Hortic. Seed 23, 162–166.

[B31] Zhang K. Wu Z. Tang D. Lv C. Luo K. Zhao Y. . (2016). Development and identification of SSR markers associated with starch properties and β-carotene content in the storage root of sweetpotato (*Ipomoea batatas* L.). Front. Plant Sci. 7, 223. doi: 10.3389/fpls.2016.00223, PMID: 26973669 PMC4773602

[B32] Zhao Z. M. (2022). Study and application of growth days and room temperature storage days in regulating the nutritional and commercial characteristics of fresh-eating sweetpotato (Henan University: Henan University of Science and Technology).

[B33] Zhong Y. Y. Cui J. C. Yu J. J. Wu X. X. Hong D. Z. Zheng J. Y. (2023). Variation laws of major nutritional components during growth of storage root in the new high carotene sweetpotato variety “Pushu 17. Anhui Agric. Sci. Bull. 29, 26–29.

